# Advanced investing with deep learning for risk-aligned portfolio optimization

**DOI:** 10.1371/journal.pone.0330547

**Published:** 2025-08-19

**Authors:** Minh Duc Nguyen

**Affiliations:** Department of Economic Information Systems, University of Economics, Hue University, Hue, Vietnam; SKEMA Business School, Universite Cote d'Azur, FRANCE

## Abstract

This study introduces a deep learning-based framework for portfolio optimization tailored to different investor risk preferences. We combine two prediction models, Long Short-Term Memory (LSTM) and One-Dimensional Convolutional Neural Network (1D-CNN), with three portfolio frameworks: Mean-Variance with Forecasting (MVF), Risk Parity Portfolio (RPP), and Maximum Drawdown Portfolio (MDP). Each framework represents a distinct risk profile: return-seeking, moderate-risk and conservative. The dataset is constructed from daily returns of VN-100 stocks in Vietnam, covering the period from 2017 to 2024. Forecasts from the deep learning models are integrated into each optimization approach. Results from the 2023–2024 test period showed that LSTM outperforms 1D-CNN in both accuracy and stability. Portfolios using LSTM achieved better performance. LSTM+MVF delivers the best risk-adjusted returns, while LSTM+MDP achieves the highest total return. The study highlights the value of aligning predictive models with appropriate optimization strategies for improved investment outcomes. Future work may include other asset classes, transaction cost modeling, and dynamic rebalancing. Combining deep learning with macroeconomic or alternative data could also improve forecasting and portfolio outcomes.

## Introduction

Portfolio optimization has long been a central topic in finance, dating back to the foundational work of Markowitz (1952) [1], who introduced the mean-variance (MV) model to balance expected return against risk. While this framework remains influential, its reliance on historical average returns and constant covariances limits its responsiveness in volatile or fast-changing markets [2]. In recent years, the integration of forecasting techniques, particularly those driven by machine learning, has emerged as a powerful enhancement to traditional portfolio theory [3–5].

Among various predictive techniques, deep learning models such as Long Short-Term Memory (LSTM) networks and One-Dimensional Convolutional Neural Networks (1D-CNN) have gained traction due to their ability to capture temporal patterns and non-linear dependencies in financial time series [6–8]. These models have demonstrated superior performance over traditional statistical methods such as ARIMA and Prophet in return prediction tasks, particularly in high-volatility and noisy market conditions. While newer architectures such as N‑BEATS offer strong performance and explicit interpretability through basis-expansion blocks [9], they are more computationally intensive and require careful tuning [10]. Similarly, attention‑based LSTM networks improve transparency in feature attribution but introduce additional complexity and parameter overhead compared to standard LSTM models [11]. In this study, we focus on LSTM and 1D-CNN due to their proven effectiveness in financial forecasting and their balance between predictive performance and model complexity, which has been validated in several prior works [5,8]. Building on this progress, recent studies have begun incorporating predicted returns into portfolio optimization frameworks, resulting in what is known as Mean-Variance with Forecasting (MVF) models [5,12]. These models extend the classical MV theory by replacing historical return estimates with model-based forecasts, and in some cases, by introducing prediction reliability as an additional optimization objective. Parallel to this development, risk-based strategies such as Risk Parity Portfolio (RPP) have emerged as viable alternatives for investors seeking balanced risk exposure without relying on potentially unstable return forecasts [13]. RPP focuses on equalizing the marginal contribution of each asset to total portfolio risk, offering a stable diversification method under volatile market conditions. For highly conservative investors, Maximum Drawdown Portfolio (MDP) models, originally proposed by Chekhlov et al. (2005) [14], provide direct control over downside risk by minimizing the worst-case loss over a specified horizon, a metric that resonates more intuitively with investor psychology than variance-based measures.

In this study, we strategically select three portfolio optimization frameworks: MVF, RPP and MDP, each representing a distinct investor risk profile. MVF serves the needs of return-maximizing investors who accept higher volatility, RPP reflects a balanced approach for moderate-risk investors, and MDP is aligned with conservative investors focused on capital preservation. These strategies are coupled with deep learning-based return forecasts from LSTM and 1D-CNN models to evaluate how predictive model quality and optimization philosophy jointly influence investment outcomes. Using a dataset constructed from the VN-100 Index in Vietnam, an emerging market with dynamic investor behavior and increasing institutional participation, we conduct a comparative analysis of these six strategy combinations. By doing so, we contribute empirical evidence on the practical value of combining data-driven forecasting with portfolio design tailored to investor-specific risk preferences.

The remainder of this paper is as follows: The next section reviews relevant literature on deep learning applications in financial forecasting and portfolio optimization techniques, with an emphasis on investor risk preferences. Following that, we present the research methodology, including data preprocessing, prediction model design, and the formulation of the three portfolio optimization strategies. The subsequent section reports and discusses the experimental results, highlighting the performance of each strategy under different forecasting models. Finally, the paper concludes by summarizing key findings, discussing practical implications, and outlining directions for future research.

## Related work

### Portfolio optimization strategies aligned with investor risk preferences

The MV framework introduced by Markowitz (1952) [1] has provided the foundation for modern portfolio theory, enabling investors to make informed decisions by balancing expected return and portfolio risk. Over time, a variety of extensions to this classical model have been developed to support more flexible investment strategies and to reflect the diverse risk preferences of different types of investors. One such extension is the MVF model, which incorporates predicted returns and average prediction errors from data-driven forecasting techniques into the portfolio optimization process [5]. MVF is particularly suitable for return-oriented investors, as it enables the construction of portfolios based on forward-looking expectations rather than historical averages, while also accounting for the reliability of those forecasts.

For investors seeking balanced exposure to risk without reliance on return forecasting, the RPP provides an alternative. RPP equalizes the marginal contribution of each asset to the portfolio’s total volatility, thereby achieving diversification through risk allocation rather than capital allocation [13]. This strategy is well aligned with moderate-risk investor preferences and has gained popularity for its robustness in uncertain market environments. At the conservative end of the spectrum, the MDP aims to minimize the maximum cumulative loss from peak to trough within a specified time horizon. Proposed by Chekhlov et al. (2005) [14], the MDP approach focuses on downside protection and tail-risk mitigation. It provides an intuitive risk measure and is especially attractive to investors prioritizing capital preservation during volatile or adverse market conditions.

### Deep learning in financial forecasting and portfolio optimization

In recent years, deep learning has emerged as a powerful tool for financial forecasting, particularly in the context of stock return prediction. Unlike traditional time series models such as ARIMA or GARCH, deep learning methods are capable of capturing complex nonlinear patterns and long-term temporal dependencies in high-frequency and noisy financial data. These capabilities make them well-suited for predicting asset returns in dynamic and volatile markets.

Among the various deep learning architectures, LSTM networks have shown particular promise in modeling sequential financial data. As a variant of recurrent neural networks (RNNs), LSTM is designed to overcome the vanishing gradient problem by introducing memory cells that retain information over extended time intervals. This feature allows LSTM networks to learn both short- and long-term dependencies in time series data, which is essential for forecasting asset returns where past events may influence future movements. Studies such as Fischer et al. (2018) and Mehtab et al. (2020) [6,8] have demonstrated the effectiveness of LSTM in predicting stock prices and returns with greater accuracy than conventional machine learning methods. Another widely used architecture in financial prediction tasks is the 1D-CNN. Originally developed for pattern recognition in image and signal processing, 1D-CNN has been successfully adapted for time series applications due to its ability to detect local features through convolutional filters. In financial contexts, 1D-CNN models can identify short-term trends, abrupt changes, and repetitive patterns in return sequences, offering a complementary perspective to LSTM models. Empirical studies have confirmed the competitive performance of 1D-CNNs in financial forecasting, particularly when integrated into hybrid architectures or used for feature extraction [7,15].

Beyond these models, recent advances in deep learning have introduced architectures such as attention mechanisms and transformer-based models, which enable dynamic weighting of input sequences and improve the model’s ability to focus on relevant historical patterns. Transformer-based models, originally developed for natural language processing, have shown strong performance in financial forecasting tasks by capturing both long- and short-term dependencies more effectively than traditional RNN-based models. Studies such as Nayak et al. (2024) and Mozaffari et al. (2024) [16,17] reported promising results using transformer variants for volatility and return prediction in financial markets, as well as for general time series forecasting tasks. In addition, N-BEATS, a deep learning architecture specifically designed for time series forecasting, has gained attention for its interpretable structure and competitive forecasting accuracy without requiring extensive preprocessing or feature engineering. Oreshkin et al. (2019) [9] demonstrated that N-BEATS achieved state-of-the-art performance on several forecasting benchmarks, including financial time series. These models represent the latest developments in machine learning for stock market prediction and highlight the growing diversity of techniques available for financial forecasting.

The integration of financial forecasting models into portfolio optimization frameworks represents a key advancement in data-driven asset allocation. While traditional optimization approaches often rely on historical average returns, recent research emphasizes the value of incorporating predicted returns, particularly those generated by machine learning models, to create forward-looking portfolios that adapt to market dynamics [18]. The effectiveness of integrating forecasts into optimization depends not only on prediction accuracy but also on how the information is utilized. Empirical studies have shown that forecast-based strategies, when paired with appropriate optimization logic, can outperform purely historical models [19]. Moreover, the use of prediction uncertainty, such as confidence intervals or historical prediction error, can help mitigate the risks associated with model overfitting or unstable predictions.

The preceding review highlights significant progress in both financial forecasting and portfolio optimization. Classical portfolio theory has evolved to accommodate a spectrum of investor risk preferences through extensions such as MVF, RPP and MDP. Simultaneously, deep learning models, particularly LSTM and 1D-CNN, have demonstrated substantial improvements in stock return prediction, offering new opportunities to integrate forward-looking signals into portfolio decision-making. While existing research has successfully applied machine learning forecasts to enhance portfolio performance, most studies tend to focus on a single optimization approach, often the MV framework. There remains limited exploration into how different types of portfolio strategies, each representing unique risk preferences, perform when combined with deep learning–based return forecasts. The interaction between predictive quality and optimization strategy therefore remains an open question. Furthermore, despite the growing relevance of machine learning in finance, empirical evidence from emerging markets such as Vietnam is still scarce. Market dynamics, liquidity conditions, and data characteristics in these contexts may differ substantially from those in developed economies, potentially affecting the performance of both predictive models and allocation strategies.

To address these gaps, this study investigates the following research questions:

1. How do LSTM and 1D-CNN models perform in forecasting returns in the Vietnamese stock market?
2. How does the integration of these forecasts with MVF, RPP, and MDP portfolio optimization strategies affect portfolio performance across different investor risk preferences?
3. Which forecasting model and optimization strategy combinations deliver superior performance in terms of returns, risk-adjusted returns, and portfolio turnover?

This study aims to address these questions by conducting a comprehensive comparative analysis of three distinct portfolio optimization frameworks (MVF, RPP and MDP), each combined with forecasts generated by LSTM and 1D-CNN models. By evaluating these strategies using data from the VN-100 Index, the research offers insights into how forecasting accuracy and portfolio logic interact in practice, and how such integration supports different investor objectives in an emerging market setting.

## Research methodology

### Data collection and preparation

This study utilizes a curated dataset based on the VN-100 Index, a widely recognized benchmark that captures the performance of the top 100 publicly traded companies across Vietnam’s principal stock exchanges. As one of the most comprehensive indicators of Vietnam’s equity market, the VN-100 offers valuable insights into both market sentiment and macroeconomic trends in an emerging Asian economy experiencing rapid financial development. Compared to other major Vietnamese indices, such as the VN-30 (which includes the 30 largest blue-chip firms) or the broader VN-Index (which includes all listed firms on HOSE), the VN-100 strikes a balance between breadth and investability. It captures over 80% of total market capitalization while maintaining sufficient liquidity for institutional relevance. While many previous studies on forecasting-based portfolio optimization have concentrated on developed markets such as the U.S., Europe, or China [5,19,20], research on emerging markets like Vietnam remains limited. The Vietnamese stock market, and particularly the VN-100 Index, presents distinct challenges, including high volatility, limited liquidity, and rapidly changing market dynamics [21,22]. These factors make it difficult for models to generalize and highlight the importance of flexible, data-driven methods in such environments. By anchoring the analysis to this index, we ensure that the models are evaluated on a broad and representative subset of the market. The initial data collection involved retrieving the daily closing prices of all VN-100 constituents over an eight-year span, from January 2017 through December 2024. To ensure the integrity and consistency of the dataset, we applied a set of eligibility filters: only those stocks with complete price histories throughout the observation period were retained. Firms that were delisted, newly listed mid-period, or exhibited significant gaps in trading records were excluded. This filtering process resulted in the exclusion of 41 stocks, leaving a final selection of 59 stocks. The final sample maintains representation across major sectors such as finance, real estate, consumer goods, and industrials, and includes mostly mid- to large-cap firms. This provided a robust and consistent dataset suitable for time series prediction and portfolio optimization.

To reflect contemporary market behavior and short-term fluctuations, we adopted a sliding window technique in constructing the model inputs. Specifically, for each prediction instance, the past 30 trading days of log returns are used as the feature window, and the models are trained to predict the return for the next trading day. This technique not only captures local temporal dynamics but also reduces the computational complexity for deep learning models by keeping the input dimensions fixed and tractable. In this study, returns for each asset are forecasted independently using a univariate modeling approach. All feature values are then standardized prior to model training to improve convergence rates and enhance model stability. Standardization rescales the data to have zero mean and unit variance using the formula:


$$z=\frac{x-\mu }{\sigma }$$ (1)

where x is the original value, μ is the feature mean and σ is the standard deviation. In our study, we employed z-score standardization because it is particularly suitable for models like LSTM and 1D-CNN that are sensitive to the scale of input features but benefit from data centered around zero [23]. This normalization ensures that input features are on a comparable scale, preventing those with larger numeric ranges from disproportionately influencing the learning process. We selected z-score standardization over min-max normalization because it is less sensitive to outliers and avoids compressing the range of the majority of data due to extreme values [15,24]. This consideration is particularly important in financial time series, where outliers may reflect genuine market behaviors rather than noise. Therefore, instead of excluding outliers, we retained them and applied z-score standardization to preserve the full distribution of observed data while maintaining numerical stability during model training.

To rigorously evaluate model generalizability, we implemented a chronological dataset split that mirrors real-world forecasting workflows and prevents data leakage. The dataset was divided into three non-overlapping segments:

- The training set (2017–2021) was used to fit model parameters.
- The validation set (2022) was used for hyperparameter tuning and model selection.
- The test set (2023–2024) was reserved for final evaluation and performance reporting on unseen data.

This sequential approach preserves the temporal order of observations and safeguards against look-ahead bias, a common pitfall in financial modeling. The structure of this experimental design ensures that all models are assessed under realistic predictive conditions and that performance reflects genuine forecasting ability rather than overfitting or data leakage.

### Models for predicting stock returns

#### Long Short-Term Memory network (LSTM).

An LSTM, or Long Short-Term Memory network [25], is a specialized type of RNN designed to process and learn from sequential data such as time series, speech, or text. LSTM networks are particularly effective at capturing long-term dependencies within sequences, which is often challenging for traditional RNNs due to the vanishing gradient problem. By addressing this issue, LSTM networks enable models to retain information over longer time horizons, making them well-suited for tasks involving time series prediction, such as stock return forecasting, where past trends and temporal patterns play a crucial role in shaping future market behavior. An LSTM unit is composed of a memory cell along with three gates: the input gate, the forget gate and the output gate. These gates regulate the flow of information into, out of and within the cell, enabling the network to selectively preserve or discard information over time. Mathematically, the behavior of an LSTM cell at time step t can be described by the following equations:


$$f_{t}=\;\sigma \left(W_{f}\cdot \;\left[h_{t-1},\;x_{t}\right]+\;b_{f}\right)$$ (2)

$$i_{t}=\;\sigma \left(W_{i}\cdot \;\left[h_{t-1},\;x_{t}\right]+\;b_{i}\right)$$ (3)

$${\tilde{C}}_{t}=\;\tanh\left(W_{C}\cdot \left[h_{t-1},\;x_{t}\right]+\;b_{C}\right)$$ (4)

$$C_{t}=\;f_{t}⊙\;C_{t-1}\;+\;i_{t}\;⊙\;{\tilde{C}}_{t}$$ (5)

$$o_{t}=\;\sigma \left(W_{o}\cdot \;\left[h_{t-1},\;x_{t}\right]+\;b_{o}\right)$$ (6)

$$h_{t}=o_{t}⊙\tanh\left(C_{t}\right)$$ (7)

In these equations:

- $x_{t}$ is the input vector at time step t,
- $h_{t-1}$ is the hidden state from the previous time step,
- $\left[h_{t-1},\;x_{t}\right]$ represents the concatenation of the previous hidden state and current input vector,
- $f_{t}$, $i_{t}$, and $o_{t}$ represent the forget gate, input gate, and output gate vectors at time t, respectively, each controlling the flow of information within the LSTM unit,
- $C_{t}$ is the cell state vector at time t, while ${\tilde{C}}_{t}$ is the candidate cell state vector generated by the current input and previous hidden state,
- σ denotes the sigmoid activation function,
- tanh represents the hyperbolic tangent activation function, used to regulate the range of cell states,
- ⊙ indicates element-wise multiplication,
- $W_{f}$, $W_{i}$, $W_{o}$, $W_{C}$ are weight matrices associated with the forget, input, output and candidate cell state computations,
- $b_{f}$, $b_{i}$, $b_{o}$, $b_{C}$ are bias vectors corresponding to each gate and the candidate state and also are learnable parameters.

Together, these equations enable the LSTM unit to regulate which information is retained, forgotten, or output at each time step, allowing it to effectively capture long-term dependencies in sequential data. To optimize the forecasting performance of the LSTM model, a grid search strategy was employed to systematically evaluate different combinations of hyperparameters. This method ensures that the model achieves a suitable balance between underfitting and overfitting by selecting parameters that minimize validation loss. Table 1 summarizes the hyperparameter values tested during grid search.

**Table 1:** Grid search-based LSTM hyperparameter options.

Hyperparameter	Range
Hidden nodes	5, 10, 15, 20
Hidden layers	1, 2, 4, 8
Learning rate	0.001, 0.005, 0.01
Batch size	32, 64, 128
Dropout rate	0.1, 0.2, 0.3
Recurrent dropout rate	0.1, 0.2, 0.3
Optimizer	Adam, RMSprop
Loss function	Mean absolute error (MAE)

After extensive experimentation, the best configuration was found to consist of four hidden layers with four units per layer, a learning rate of 0.01, a batch size of 64, a dropout rate and recurrent dropout rate of 0.3, and the Adam optimizer. This configuration yielded the lowest average validation MAE and was therefore used to train the LSTM forecasting model across all stocks and generate return predictions.

#### One-Dimensional Convolutional Neural Network (1D-CNN).

CNNs, originally developed for image recognition tasks, have been successfully adapted for time-series data through a one-dimensional configuration known as 1D-CNN. Unlike traditional CNNs that operate on two-dimensional image grids, 1D-CNNs apply convolutional filters across temporal sequences, making them particularly suitable for extracting localized patterns from financial time-series such as stock returns. In the context of stock return forecasting, 1D-CNNs are designed to detect short-term trends or repeating behaviors over time by using convolutional filters that slide across sequences of daily returns. These filters act as feature extractors, capturing essential dynamics such as spikes, reversals, or momentum patterns that may influence future price movements. The extracted features are then passed through pooling layers to reduce dimensionality and subsequently to fully connected layers for prediction. The core operation in a 1D-CNN is the convolution, defined mathematically as:


$$\begin{array}{c} y_{t}=\sum {\mathrm{\backslash nolimits}}_{i=0}^{k-1}x_{t+i}\cdot w_{i}+b \end{array}$$ (8)

where:

- $y_{t}$ is the output of the convolutional layer at time t,
- i is the index variable ranging from 0 to k−1, where k is the kernel size,
- $x_{t+i}$ is the input at time step t+i,
- $w_{i}$ is the weight of the kernel/filter at position i,
- b is the scalar bias term.

This operation is applied repeatedly as the filter “slides” across the input sequence. Multiple filters can be used to capture different types of patterns in parallel. ReLU (Rectified Linear Unit) activation is typically applied after each convolution to introduce non-linearity. Pooling layers (e.g., max pooling) follow the convolution layers to reduce overfitting and computation by downsampling the feature maps. The resulting lower-dimensional representations are then flattened and passed through one or more dense layers to produce the final prediction. Similar to our approach with LSTM in this study, a grid search was conducted to tune the hyperparameters of the 1D-CNN. Table 2 summarizes the range of hyperparameter values searched.

**Table 2:** Grid search-based 1D-CNN hyperparameter options.

Hyperparameter	Range
Number of Filters	32, 64, 128
Kernel Size	3, 5, 7
Number of Conv Layers	1, 2, 3
Pooling Type	Max, Average
Pooling Size	2, 4
Dropout Rate	0.0, 0.2, 0.5
Dense Layer Units	32, 64, 128
Activation	ReLU, Tanh
Optimizer	Adam, RMSprop
Learning Rate	0.0001, 0.05, 0.01
Batch Size	32, 64, 128
Epochs	50, 100

Through comprehensive experimentation, the most effective 1D-CNN architecture was identified as consisting of two convolutional layers with 32 filters each and a kernel size of 3, followed by a max pooling layer with a pooling size of 2, ReLU activation, and a dense layer with 64 units. The model used a dropout rate of 0.2, was trained with a batch size of 64 over 50 epochs, and optimized using the Adam optimizer with a learning rate of 0.01. This setup produced the lowest average validation MAE and was adopted to stock return prediction in this study.

### Frameworks for optimizing investment portfolios

#### Return-focused investing with Mean-Variance with Forecasting (MVF).

Markowitz (1952) [1] introduced MV portfolio theory which established the foundation of modern investment strategy by proposing that investors should select portfolios based on the optimal trade-off between expected return and risk. His formulation introduced the concept of the efficient frontier and demonstrated that asset selection should consider not only individual return and risk characteristics but also the covariances among assets. To enhance the responsiveness of the MV framework in dynamic market conditions, this study adopts the MVF model proposed by Yu et al. (2020) and Ma et al. (2021) [5,12]. The MVF model extends the classical MV theory by integrating forecasted returns and prediction reliability directly into the optimization objective. Specifically, instead of relying on historical average returns, MVF incorporates return forecasts (e.g., from LSTM or 1D-CNN) and additionally accounts for the confidence level in these predictions by treating average predictive errors as indicators of abnormal returns. The MVF model is formulated as follows:


$$\begin{array}{c} \min\left(\sum {\mathrm{\backslash nolimits}}_{i=1}^{n}\sum {\mathrm{\backslash nolimits}}_{j=1}^{n}x_{i}x_{j}\sigma_{ij}-\lambda \left(\sum {\mathrm{\backslash nolimits}}_{i=1}^{n}x_{i}{\hat{r}}_{i}+\sum {\mathrm{\backslash nolimits}}_{i=1}^{n}x_{i}{\overset{―}{\varepsilon }}_{i}\right)\right) \end{array}$$ (9)

subject to:


$$\begin{array}{c} \sum {\mathrm{\backslash nolimits}}_{i=1}^{n}x_{i}=1\;\text{and}\;0\leq x_{i}\leq 1\;\text{for}\;i=1,2,\ldots ,n \end{array}$$ (10)

where:

- $x_{i}$ is the proportion of asset i in the portfolio,
- $\sigma_{ij}$ is the covariance between the returns of asset i and asset j,
- λ is a nonnegative user-defined parameter that reflects the investor’s preference between risk and returns (λ=0 indicates complete risk aversion, while higher values imply greater return-seeking behavior),
- ${\hat{r}}_{i}$ is the forecasted return of asset i provided by LSTM or 1D-CNN,
- ${\overset{―}{\varepsilon }}_{i}$ is the average residual for asset i, representing abnormal returns based on forecast errors.

In this study, λ is set to 1 to match the original formulation and maintain balance. The MVF objective seeks to construct a portfolio that minimizes total risk while maximizing forecasted returns and potential abnormal returns. This model thus reflects a return-focused investment strategy informed by both predictive insights and risk control.

#### Moderate risk allocation with Risk Parity Portfolio (RPP).

The RPP represents a risk-based investment strategy that aims to allocate capital such that each asset contributes equally to the overall portfolio risk. Unlike traditional mean-variance approaches, which rely on estimates of expected returns, the RPP methodology focuses exclusively on the risk characteristics of assets. This makes it particularly appealing for investors with moderate risk tolerance who seek consistent diversification and reduced sensitivity to return estimation errors. The central premise of RPP is that equal capital allocations do not necessarily lead to equal risk contributions, particularly in portfolios that include assets with significantly different volatilities. Risk parity seeks to resolve this by balancing the marginal risk contribution of each asset to the portfolio’s total volatility. As emphasized by Edward Qian (2005) [13], this leads to true diversification, where no single asset disproportionately drives portfolio risk. Mathematically, the total portfolio volatility is expressed as:


$$\sigma_{p}=\sqrt{x^{T}\mathrm{\backslash sumx}}$$ (11)

where $x\in {\mathbb{R}}^{n}$ is the vector of asset weights and ∑ is the covariance matrix of asset returns. The risk contribution of asset i is defined as:


$${RC}_{i}(x)=x_{i}\cdot \frac{{\left(\mathrm{\backslash sumx}\right)}_{i}}{\sigma_{p}}$$ (12)

The objective of the risk parity framework is to equalize these contributions. A common formulation, which is also adopted in this study, involves minimizing the sum of squared differences in the risk contributions between all pairs of assets:


$$\min_{x}\sum_{i=1}^{n}\sum_{j=1}^{n}{\left({RC}_{i}(x)-{RC}_{j}(x)\right)}^{2}$$ (13)

subject to:


$$\begin{array}{c} \sum {\mathrm{\backslash nolimits}}_{i=1}^{n}x_{i}=1\;\text{and}\;0\leq x_{i}\leq 1\;\text{for}\;i=1,2,\ldots ,n \end{array}$$ (14)

Overall, the RPP strategy offers a compelling alternative for moderate-risk investors. By eliminating dependence on unstable return forecasts and focusing on risk contributions, it provides a robust mechanism for constructing balanced portfolios that are resilient across various market conditions.

#### Conservative investing through Maximum Drawdown Portfolio (MDP).

For investors with a highly conservative risk profile, managing downside exposure is a primary concern. While traditional portfolio optimization methods such as MV and risk parity frameworks focus on volatility or risk balancing, they do not explicitly address extreme loss scenarios. The MDP model is designed to directly minimize maximum drawdown, which captures the worst cumulative loss from a portfolio’s peak to its subsequent trough over a given investment horizon. Maximum drawdown is a path-dependent risk measure that reflects an investor’s aversion to significant losses more accurately than variance. Unlike standard deviation, which penalizes both upside and downside deviations, drawdown measures focus solely on negative performance, making them particularly relevant for capital preservation objectives. The foundational methodology for drawdown-based optimization was introduced by Chekhlov et al. (2005) [14], who formalized the use of maximum drawdown and Conditional Drawdown-at-Risk (CDaR) in portfolio selection. Their framework enables the direct control of downside risk through convex optimization, aligning closely with conservative investor preferences.

Let $x\in {\mathbb{R}}^{n}$ denote the portfolio weights and $R_{t}\in {\mathbb{R}}^{n}$ present the vector of asset returns at time t. The portfolio return at time t is:


$$r_{t}(x)=x^{T}R_{t}$$ (15)

The cumulative portfolio value at time t is:


$$\begin{array}{c} V_{t}(x)=\prod {\mathrm{\backslash nolimits}}_{s=1}^{t}\left(1+r_{s}(x)\right) \end{array}$$ (16)

The maximum drawdown (MDD) over period T is:


$$MDD(x)=\max_{t\in [1,T]}\left(\max_{s\in [1,t]}V_{s}(x)-V_{t}(x)\right)$$ (17)

The optimization objective for MDP is therefore:


$$\min_{x}MDD(x)$$ (18)

subject to:


$$\begin{array}{c} \sum {\mathrm{\backslash nolimits}}_{i=1}^{n}x_{i}=1\;\text{and}\;0\leq x_{i}\leq 1\;\text{for}\;i=1,2,\ldots ,n \end{array}$$ (19)

In this study, maximum drawdown is evaluated over historical return sequences informed by LSTM and 1D-CNN forecasts, allowing the optimization process to incorporate both forward-looking performance expectations and risk control. The MDP approach, by directly addressing the most adverse loss scenarios, aligns with the goals of conservative investors who prioritize capital preservation over short-term gains.

## Experimental results

### Evaluation of stock return prediction accuracy

Accurate forecasting of stock returns is a critical component in the success of predictive portfolio optimization strategies. In this study, the performance of the return prediction models is evaluated using six evaluation metrics commonly employed in time series forecasting and financial prediction. These metrics are designed to evaluate both the magnitude and directional accuracy of the predicted returns. Specifically, we report the mean absolute error (MAE), mean squared error (MSE), and root mean squared error (RMSE) to assess prediction accuracy in terms of value deviation. Additionally, we include the total hit ratio ($H_{R}$), positive-directional hit ratio ($H_{R+}$), and negative-directional hit ratio ($H_{R-}$) to measure the models’ ability to correctly predict the direction of returns. The definitions of these metrics are summarized in Table 3, where $y_{i}$ is the actual observed return, ${\hat{y}}_{i}$ is the predicted return and n is the number of observations in the test set.

**Table 3:** Evaluation metrics of stock return prediction.

Metric	Formula
Mean Absolute Error (MAE)	$\begin{array}{c} MAE=\frac{1}{n}\sum_{i=1}^{n}\left|y_{i}-{\hat{y}}_{i}\right| \end{array}$
Mean Squared Error (MSE)	$\begin{array}{c} MSE=\frac{1}{n}\sum_{i=1}^{n}{\left(y_{i}-{\hat{y}}_{i}\right)}^{2} \end{array}$
Root Mean Squared Error (RMSE)	$RMSE=\sqrt{MSE}$
Total hit ratio ($H_{R}$)	$H_{R}=\frac{{count}_{i=1}^{n}\left(y_{i}\cdot {\hat{y}}_{i}>0\right)}{{count}_{i=1}^{n}\left(y_{i}\cdot {\hat{y}}_{i}\neq 0\right)}$
Positive hit ratio ($H_{R+}$)	$H_{R+}=\frac{{count}_{i=1}^{n}\left(y_{i}>0\;and\;{\hat{y}}_{i}>0\right)}{{count}_{i=1}^{n}\left({\hat{y}}_{i}>0\right)}$
Negative hit ratio ($H_{R-}$)	$H_{R-}=\frac{{count}_{i=1}^{n}\left(y_{i}<0\;and\;{\hat{y}}_{i}<0\right)}{{count}_{i=1}^{n}\left({\hat{y}}_{i}<0\right)}$

These metrics are computed for each stock across the test horizon and then averaged across the full set of assets. Lower values of MAE, MSE and RMSE indicate better predictive performance in terms of error magnitude, while higher values of $H_{R}$, $H_{R+}$, and $H_{R-}$ reflect stronger directional accuracy. Note that this study designates MAE, MSE, and RMSE as the primary evaluation metrics, as they play a critical role in guiding portfolio construction based on return predictions. Table 4 presents the performance comparison of the LSTM and 1D-CNN models based on such metrics.

**Table 4:** Performance assessment of two models in prediction.

Prediction model		MAE	MSE	RMSE	$H_{R}$	$H_{R+}$	$H_{R-}$
LSTM	Mean	1.56 × 10^−2^	4.76 × 10^−4^	2.12 × 10^−2^	48.34%	48.00%	47.95%
	Std	3.96 × 10^−3^	2.11 × 10^−4^	5.04 × 10^−3^	2.66 × 10^−2^	3.79 × 10^−2^	3.95 × 10^−2^
1D-CNN	Mean	1.60 × 10^−2^	5.03 × 10^−4^	2.18 × 10^−2^	48.49%	48.19%	48.01%
	Std	4.12 × 10^−3^	2.29 × 10^−4^	5.31 × 10^−3^	2.56 × 10^−2^	3.89 × 10^−2^	3.37 × 10^−2^

In terms of average predictive accuracy, the LSTM model slightly outperforms the 1D-CNN model across all three magnitude-based error metrics. The mean MAE for LSTM is 1.56 × 10^−2^, compared to 1.60 × 10^−2^ for 1D-CNN, indicating that LSTM generates marginally more precise predictions on average. Similarly, the MSE values suggest that LSTM better controls large forecast errors, with a lower mean MSE of 4.76 × 10^−4^ relative to 5.03 × 10^−4^ for 1D-CNN. This advantage is further reflected in the RMSE results, where LSTM achieves a value of 2.12 × 10^−2^, slightly lower than 2.18 × 10^−2^ for 1D-CNN. Additionally, LSTM exhibits lower standard deviations in MAE, MSE, and RMSE, reflecting greater stability and reliability across different stocks and time periods. Beyond magnitude accuracy, directional prediction is another key consideration. The results show that both models perform similarly in directional forecasting. While the differences are marginal, they suggest that both models are similarly effective in capturing the direction of return movements under varying market conditions.

In summary, LSTM demonstrates better performance in minimizing forecast errors and exhibits more consistent behavior across evaluation metrics, making it a more robust choice for return prediction. Although directional metrics show only slight differences between the two models, LSTM’s superior performance in error-based evaluation and its lower prediction variability reinforce its suitability for integration into portfolio optimization frameworks.

### Evaluation of portfolio optimization strategies

To comprehensively assess the effectiveness of the proposed portfolio optimization frameworks, this section evaluates six strategy combinations that integrate two predictive models of LSTM and 1D-CNN with three optimization frameworks including MVF, RPP and MDP. The portfolio is rebalanced monthly (approximately every 20–22 trading days), in line with standard practice in empirical portfolio optimization studies [12]. For performance benchmarking, we assume no transaction costs and evaluate each strategy’s monthly excess return relative to the VN-100 Index over the test period from 2023 to 2024. The comparison includes the mean and standard deviation of monthly excess returns, information rate, total return as well as turnover rate, allowing for an in-depth assessment of how effectively each strategy balances risk and return. By referencing the VN-100, a representative index of the Vietnamese equity market, we contextualize the outcomes of each strategy against a relevant market benchmark, thereby enhancing interpretability of the results within a real-world investment setting. While transaction costs are excluded from this analysis for simplicity, it is important to recognize that frequent portfolio rebalancing may lead to substantial trading costs in practice. Such costs can reduce net returns and limit the real-world applicability of high-turnover strategies. To address this, we report the portfolio turnover rates alongside the performance metrics to provide additional insight into the trading intensity of each strategy. This enables a more comprehensive evaluation of the trade-offs between return potential and practical implementability. Table 5 presents the performance comparison of the six portfolio strategies.

**Table 5:** Performance assessment of six strategies.

Strategy	Monthly excess return		Information rate	Total return (%)	Turnover rate (%)
	Mean (%)	Std			
LSTM+MVF	2.208	0.042	0.526	50.775	80.878
1D-CNN + MVF	1.978	0.041	0.479	45.499	80.236
LSTM+RPP	1.480	0.055	0.268	34.034	54.137
1D-CNN + RPP	1.471	0.056	0.265	33.824	60.224
LSTM+MDP	2.281	0.046	0.499	52.459	78.697
1D-CNN + MDP	1.473	0.059	0.250	33.877	55.454

Among the strategies, LSTM+MDP achieves the highest total return of 52.459%, closely followed by LSTM+MVF with 50.775%. These results suggest that LSTM-based forecasts contribute more effectively to both capital preservation and growth compared to 1D-CNN. Notably, LSTM+MDP also yields a strong monthly excess return mean of 2.281% and an information ratio of 0.499, indicating its strength not only in raw performance but also in consistency relative to risk. Its turnover rate remains relatively high at 78.697%, which reflects more frequent rebalancing. The LSTM+MVF strategy shows a slightly lower return than LSTM+MDP but achieves the highest information ratio (0.526) among all strategies, suggesting strong risk-adjusted performance, with the highest turnover rate of 80.878%. To further examine the difference between these two top-performing strategies, we conducted a Mann-Whitney test on their monthly excess returns. The test results showed no statistically significant difference (p = 0.265), suggesting that the observed performance differences between LSTM+MDP and LSTM+MVF may not be statistically meaningful. Meanwhile, 1D-CNN+MVF trails its LSTM-based counterpart in both total return (45.499%) and information ratio (0.479), with a similarly high turnover rate of 80.236%, highlighting the advantage of LSTM in capturing time-series dynamics for return prediction. The RPP-based strategies, although more stable in terms of volatility, generate lower performance overall. LSTM+RPP and 1D-CNN+RPP report nearly identical results, with total returns of 34.034% and 33.824%, respectively, and information ratios of approximately 0.26, and moderate turnover rates of 54.137% and 60.224%, respectively. These findings align with the RPP framework’s objective of balanced risk allocation rather than aggressive return maximization. Interestingly, 1D-CNN+MDP, despite sharing the same conservative philosophy as its LSTM counterpart, underperforms significantly, yielding the lowest total return (33.877%) and the lowest information ratio (0.250), and a relatively moderate turnover rate of 55.454%. This suggests that MDP performance is highly sensitive to the accuracy and reliability of return forecasts, where LSTM consistently demonstrates superiority over 1D-CNN.

Overall, the results support the effectiveness of combining deep learning-based forecasts with portfolio optimization methods tailored to specific risk preferences. LSTM-driven strategies consistently outperform their 1D-CNN counterparts, and MVF and MDP each provide advantages for return-focused and conservative investors, respectively. The findings confirm that aligning predictive and optimization components with investor profiles enhances both return potential and risk-adjusted performance.

In addition, to rigorously assess whether the portfolio strategies achieved statistically significant outperformance relative to the VN-100 index, we conducted formal hypothesis tests on the excess returns of each portfolio. First, we applied the Shapiro-Wilk test to examine the normality of the excess return distributions. The results indicated that all portfolios exhibited non-normal return distributions. Consequently, we employed the non-parametric Wilcoxon signed-rank test to evaluate whether the median excess returns were significantly greater than zero. The test results revealed that all six portfolios achieved statistically significant outperformance with p-values below 0.05. These findings confirm that the returns of all strategies were not only higher than the benchmark on average but also statistically significant, reinforcing the robustness of the proposed predictive portfolio optimization frameworks.

**Fig 1:**
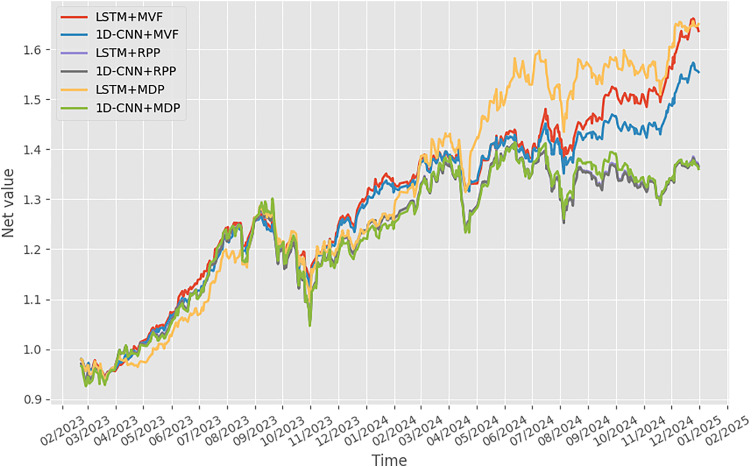
Comparative net gains across strategies.

To further illustrate the comparative performance of the six strategies, Fig 1 plots the cumulative net value of each portfolio over the evaluation period. This visual representation captures the trajectory of wealth accumulation under each strategy, highlighting not only differences in final returns but also the evolution of gains and drawdowns over time.

Specifically, the LSTM+MDP strategy demonstrates superior performance in terms of consistent upward growth, achieving the highest final net value by the end of the test period. The LSTM+MVF strategy also shows strong performance, closely tracking MDP in the latter half of the period, albeit with more noticeable volatility. In contrast, the RPP-based strategies (both LSTM and 1D-CNN) exhibit relatively flat trajectories with smaller gains, aligning with their design goal of maintaining moderate risk exposure and balanced asset contributions. Notably, 1D-CNN-based strategies generally underperform their LSTM counterparts, with 1D-CNN+MDP showing significant drawdowns in the second half of 2024. This further supports the earlier observation that LSTM provides more stable and effective return forecasts, especially when integrated into optimization frameworks sensitive to extreme downside risk, such as MDP.

**Fig 2:**
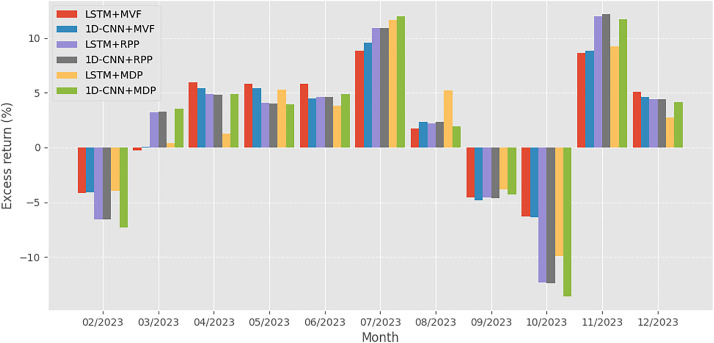
Strategy-specific excess returns recorded in 2023.

**Fig 3:**
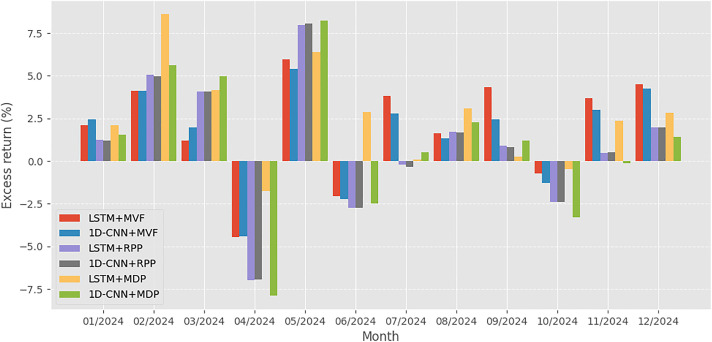
Strategy-specific excess returns recorded in 2024.

To complement the cumulative net value trajectories shown in Fig 1, Figs 2 and 3 provide a month-by-month breakdown of each strategy’s excess return during the years 2023 and 2024, respectively. These figures allow for a more granular examination of how each model-optimization combination performed across different market conditions.

Overall, the results in Figs 1–3 reinforce the insights derived from Table 5, visually confirming that the combination of deep learning-based forecasting and portfolio strategies tailored to investor risk preferences leads to distinct performance outcomes. The most favorable results are achieved when forecasting quality (LSTM) is matched with an optimization strategy aligned with the investor’s objectives, whether it is maximizing return (by using MVF) or minimizing risk (by using MDP).

## Conclusion

This study presents a comprehensive framework that integrates deep learning-based return prediction models with tailored portfolio optimization strategies to support risk-aligned investment decision-making. By combining LSTM and 1D-CNN models with three distinct portfolio construction approaches (MVF, RPP and MDP), the proposed methodology accommodates a spectrum of investor risk preferences ranging from aggressive to conservative. Empirical results based on data from Vietnam’s VN-100 Index during 2023–2024 show that LSTM consistently outperforms 1D-CNN in terms of both predictive accuracy and model stability. This superior forecasting capability translates into improved investment performance across all portfolio strategies, particularly in return-driven (MVF) and risk-sensitive (MDP) configurations. Among the tested combinations, the LSTM+MDP strategy achieved the highest total return, while LSTM+MVF delivered the best risk-adjusted performance based on the information ratio. Conversely, 1D-CNN-based portfolios exhibited lower overall returns and greater sensitivity to forecast errors, reaffirming the critical role of reliable prediction inputs in data-driven asset allocation. Although the deep learning models in this study are trained exclusively on historical return sequences, the patterns they learn may implicitly reflect underlying economic phenomena such as momentum effects, volatility clustering, or investor sentiment. These market behaviors often manifest in price dynamics and can be captured by the temporal structures in the data. For instance, LSTM’s ability to model long-term dependencies may make it sensitive to sustained macroeconomic cycles or persistent structural shifts in the market. While these economic factors are not explicitly modeled in our framework, their influence may be embedded within the learned representations.

From a practical perspective, the findings underscore the value of aligning forecasting models with portfolio construction techniques that reflect investor objectives. Incorporating model confidence (via prediction error) into the optimization process, as demonstrated in the MVF formulation, further enhances decision robustness. The study also affirms the viability of adopting deep learning frameworks in emerging markets such as Vietnam, where data quality and market dynamics present both challenges and opportunities. The results of this study offer practical guidance for investors seeking to align their portfolio strategies with both market conditions and personal risk profiles. For institutional investors with higher capital bases and greater tolerance for complexity and turnover, the LSTM+MVF strategy may be particularly attractive due to its superior risk-adjusted performance, despite its higher rebalancing intensity. In contrast, highly risk-averse individual investors may benefit more from the LSTM+MDP strategy, which provides stronger downside protection and capital preservation through maximum drawdown minimization. Meanwhile, moderate-risk investors, whether individuals or institutions, could consider RPP-based strategies for their stability, though they may trade off some return potential. Across all cases, our findings reinforce the importance of matching predictive modeling capabilities with optimization frameworks that reflect investor-specific goals and constraints. In practical settings, this may involve adjusting the frequency of rebalancing or incorporating simplified versions of the strategies for broader accessibility. It is important to note that this study focuses exclusively on forecasting expected returns, following common practice in prior portfolio optimization research. Although joint forecasting of both returns and covariances could potentially improve optimization outcomes, such an approach is beyond the scope of this study and is left for future research.

Future research may expand on this work by incorporating additional datasets from other markets to assess the robustness and generalizability of the proposed approach, and to determine whether the observed results are specific to Vietnam’s equity market or consistent across different financial environments. Extending the analysis to other asset classes (e.g., bonds or commodities) could further broaden the applicability of the framework. Introducing transaction cost modeling or extending the framework to multi-period and dynamic portfolio rebalancing, where portfolio decisions explicitly consider the path and long-term effects of rebalancing strategies, would help align the approach more closely with practical investment conditions. Moreover, incorporating traditional econometric models as benchmarks could offer valuable comparisons and help bridge the gap between deep learning-based methods and conventional approaches commonly used in financial research. In addition, future studies could address the interpretability of deep learning models by examining the underlying economic drivers of predictions, such as momentum, volatility, or other market-based factors. Applying explainable AI techniques (e.g., SHapley Additive exPlanations) could provide insights into feature importance and enhance the transparency of deep learning-driven portfolio decisions.

## Supporting information

S1 Dataset Daily closing prices of stocks within the VN-100 index (2017–2024). (CSV)
